# Supplementary material for: Environmental regulation of toxin production in Bacillus anthracis
Source: PLoS Pathog. 2025 Dec 1;21(12):e1013587. doi: 10.1371/journal.ppat.1013587 (PMC12680359; doi:10.1371/journal.ppat.1013587)
Supplement: S1 Fig — Up-regulated pathways (NES > 0) are colored red, while down-regulated pathways (NES < 0) are colored blue. B) Representative enrichment score for the PTS pathways (bar02060_PTS). Vertical bars represent the grouping pattern of individual genes of the PTS pathway based on their net enrichment scores. The heatmap below indicates the leading genes identified based on their enrichment scores. C) Ridge plots representing significantly dysregulated metabolic pathways in B. anthracis Wt grown under toxin-producing conditions (glucose + 5% CO2) versus non-toxin-producing conditions (no glucose + 5% CO2). Up-regulated pathways (NES > 0) are colored red, while down-regulated pathways (NES < 0) are colored blue. D) Representative enrichment score for the PTS pathways (bar02060_PTS). Vertical bars represent the grouping pattern of individual genes of the PTS pathway based on their net enrichment scores. The heatmap below indicates the leading genes identified based on their enrichment scores. (DOCX) [file ppat.1013587.s001.docx]

**Supplementary Figure- 1.**

**

**

**A)** Ridge plots representing significantly dysregulated metabolic pathways in *B. anthracis* Wt grown under toxin-producing conditions (glucose + 5% CO_2_) versus non-toxin-producing conditions (glucose + air). Up-regulated pathways (NES > 0) are colored red, while down-regulated pathways (NES < 0) are colored blue.

**B)** Representative enrichment score for the PTS pathways (bar02060_PTS). Vertical bars represent the grouping pattern of individual genes of the PTS pathway based on their net enrichment scores. The heatmap below indicates the leading genes identified based on their enrichment scores.

**C)** Ridge plots representing significantly dysregulated metabolic pathways in *B. anthracis* Wt grown under toxin-producing conditions (glucose + 5% CO_2_) versus non-toxin-producing conditions (no glucose + 5% CO_2_). Up-regulated pathways (NES > 0) are colored red, while down-regulated pathways (NES < 0) are colored blue.

**D)** Representative enrichment score for the PTS pathways (bar02060_PTS). Vertical bars represent the grouping pattern of individual genes of the PTS pathway based on their net enrichment scores. The heatmap below indicates the leading genes identified based on their enrichment scores.
