# Supplementary material for: Environmental regulation of toxin production in Bacillus anthracis
Source: PLoS Pathog. 2025 Dec 1;21(12):e1013587. doi: 10.1371/journal.ppat.1013587 (PMC12680359; doi:10.1371/journal.ppat.1013587)
Supplement: S2 Fig — The color gradient (red to blue) indicates fold change in metabolite levels compared to air and no-glucose conditions. Bubble size represents the p-value as -log(p). B) Scatter plot of selected glycolysis (black dots) and TCA (grey dots) intermediates comparing several growth conditions. Growth conditions for each comparison are mentioned at the top of each graph. The comparisons are made as follows: Variable-1 vs Variable-2 (constant). The x-axis indicates fold change in metabolite levels and y-axis represents the p-value as -log(p). (DOCX) [file ppat.1013587.s002.docx]

**S2 Figure.**

**
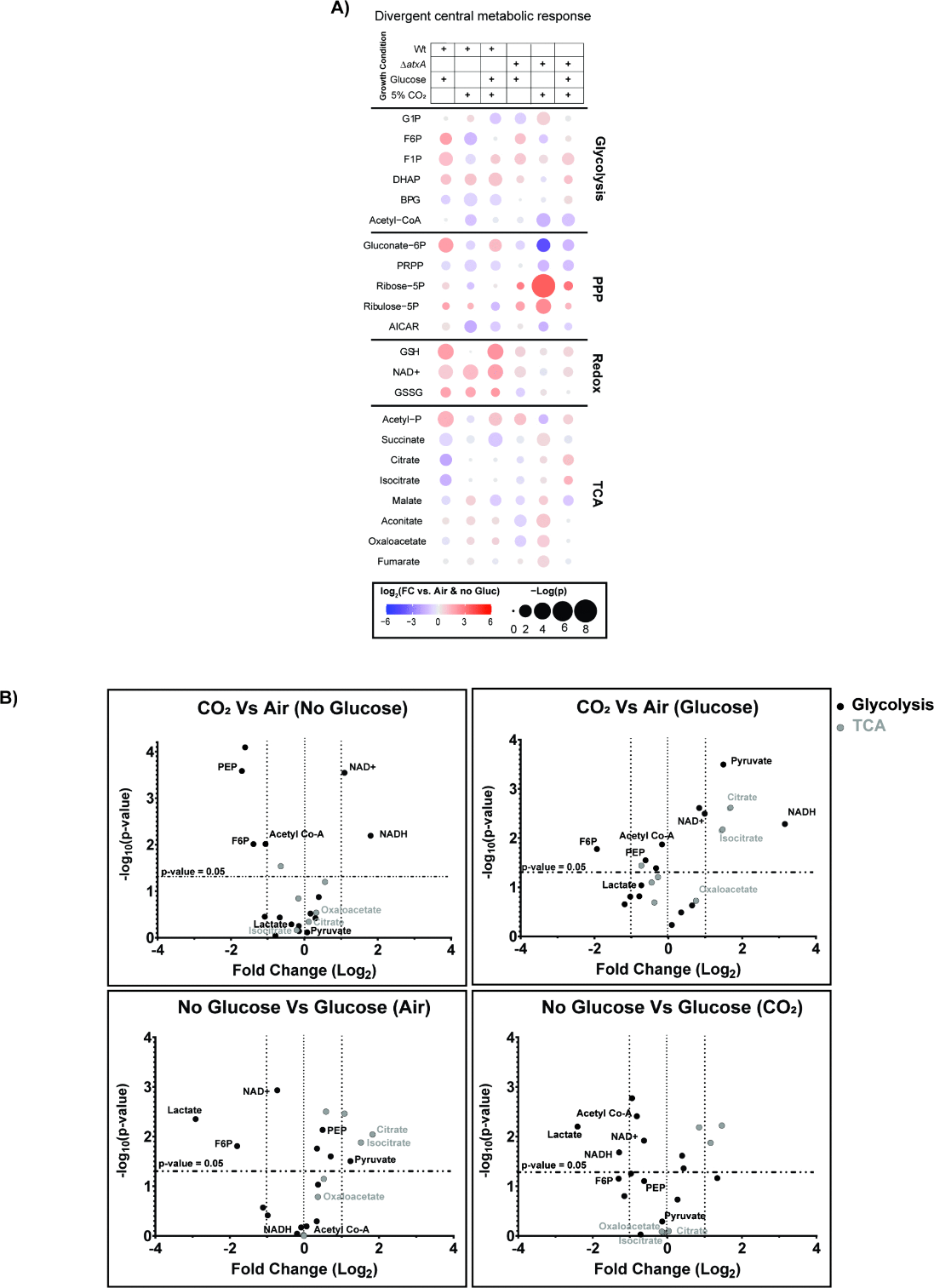
**

**A)** Bubble plot showing the relative abundance of divergent carbon metabolism metabolites significantly dysregulated under different growth conditions in *B. anthracis* Wt and *ΔatxA*. The color gradient (red to blue) indicates fold change in metabolite levels compared to air and no-glucose conditions. Bubble size represents the p-value as -log(p).

**B)** Scatter plot of selected glycolysis (black dots) and TCA (grey dots) intermediates comparing several growth conditions. Growth conditions for each comparison are mentioned at the top of each graph. The comparisons are made as following: Variable-1 vs Variable-2 (constant). The x-axis indicates fold change in metabolite levels and y-axis represents the p-value as -log(p).
