# Supplementary material for: Environmental regulation of toxin production in Bacillus anthracis
Source: PLoS Pathog. 2025 Dec 1;21(12):e1013587. doi: 10.1371/journal.ppat.1013587 (PMC12680359; doi:10.1371/journal.ppat.1013587)
Supplement: S3 Fig — Lanes are marked from 1-7 for the list of strains and growth conditions used in the table below. M = Protein molecular ladder. B,C) Bar graphs showing the absolute abundance (y-axis) of peptides for respective proteins (Uniprot IDs, x-axis) identified from co-affinity purification samples. Cyan bars indicate abundance in the pulled-down sample, while red bars indicate background or non-specific proteins. Each protein is classified based on its molecular function in the table below. B) AtxA interactions identified from bacteria grown in air. C) AtxA interactions identified from bacteria grown in 5% CO2. Glucose was present in both conditions. (DOCX) [file ppat.1013587.s003.docx]

**S3 Fig.**

**
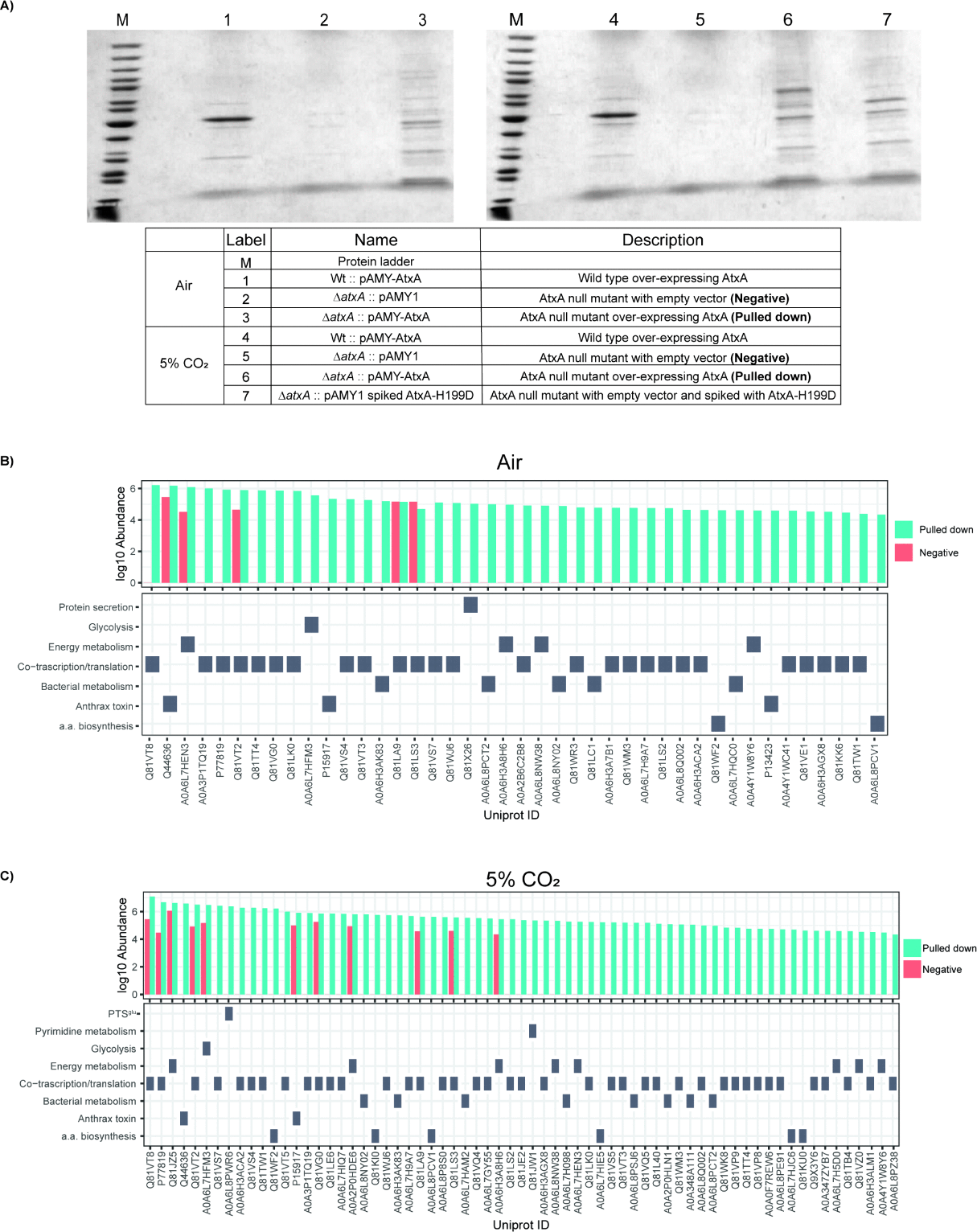
**

**A)** Coomassie-stained polyacrylamide gel of the elutes from co-affinity samples. Lanes are marked from 1-7 for the list of strains and growth conditions used in the table below. M = Protein molecular ladder.

**B,C)** Bar graphs showing the absolute abundance (y-axis) of peptides for respective proteins (Uniprot IDs, x-axis) identified from co-affinity purification samples. Cyan bars indicate abundance in the pulled-down sample, while red bars indicate background or non-specific proteins. Each protein is classified based on its molecular function in the table below. B) AtxA interactions identified from bacteria grown in air. C) AtxA interactions identified from bacteria grown in 5% CO_2_. Glucose was present in both conditions.
