# Supplementary material for: Environmental regulation of toxin production in Bacillus anthracis
Source: PLoS Pathog. 2025 Dec 1;21(12):e1013587. doi: 10.1371/journal.ppat.1013587 (PMC12680359; doi:10.1371/journal.ppat.1013587)
Supplement: S4 Fig — Supernatant volumes were normalized against total cellular protein for comparative analysis. Representative graph of n = 2 experiments. B) Growth kinetics of all B. anthracis strains under toxin-producing conditions (glucose + 5% CO2). Each data point represents the average of three replicates. Representative image of n = 2 experiments. C) Heatmap showing the relative abundance of significantly altered metabolites in ΔptsG, Δpyc, and ΔptsG/Δpyc mutants compared to their respective abundance in B. anthracis WT strain grown under toxin-producing conditions (glucose + 5% CO2). The left heatmap shows the log2 fold change in abundance as a color gradient from blue (low abundance) to red (high abundance). The right heatmap corresponds to the p-value of each data point on the left heatmap, represented as a color gradient from grey (p > 0.1, insignificant) to orange (p = 0-0.01, highly significant). D) Representative FRET-FLIM based interactions in strains expressing different FRET partners. The violin/distribution plot represents the median GFP decay profile (in nanoseconds, ns) GFP variant C4 in the respective strains. The graph represents n = 2 experiments, * indicates p-value. E) Immunoblotting for PagA showing effects of loss of the EIIB domain of AtxA (AtxA-ΔEIIB-C4) on anthrax toxin expression. Having AtxA variants expressed in the background of B. anthracis ΔatxA strains, with plasmid pAMY expressing the respective AtxA variants. Representative image of n = 3 experiments. (DOCX) [file ppat.1013587.s004.docx]

**S4 Fig.**

**
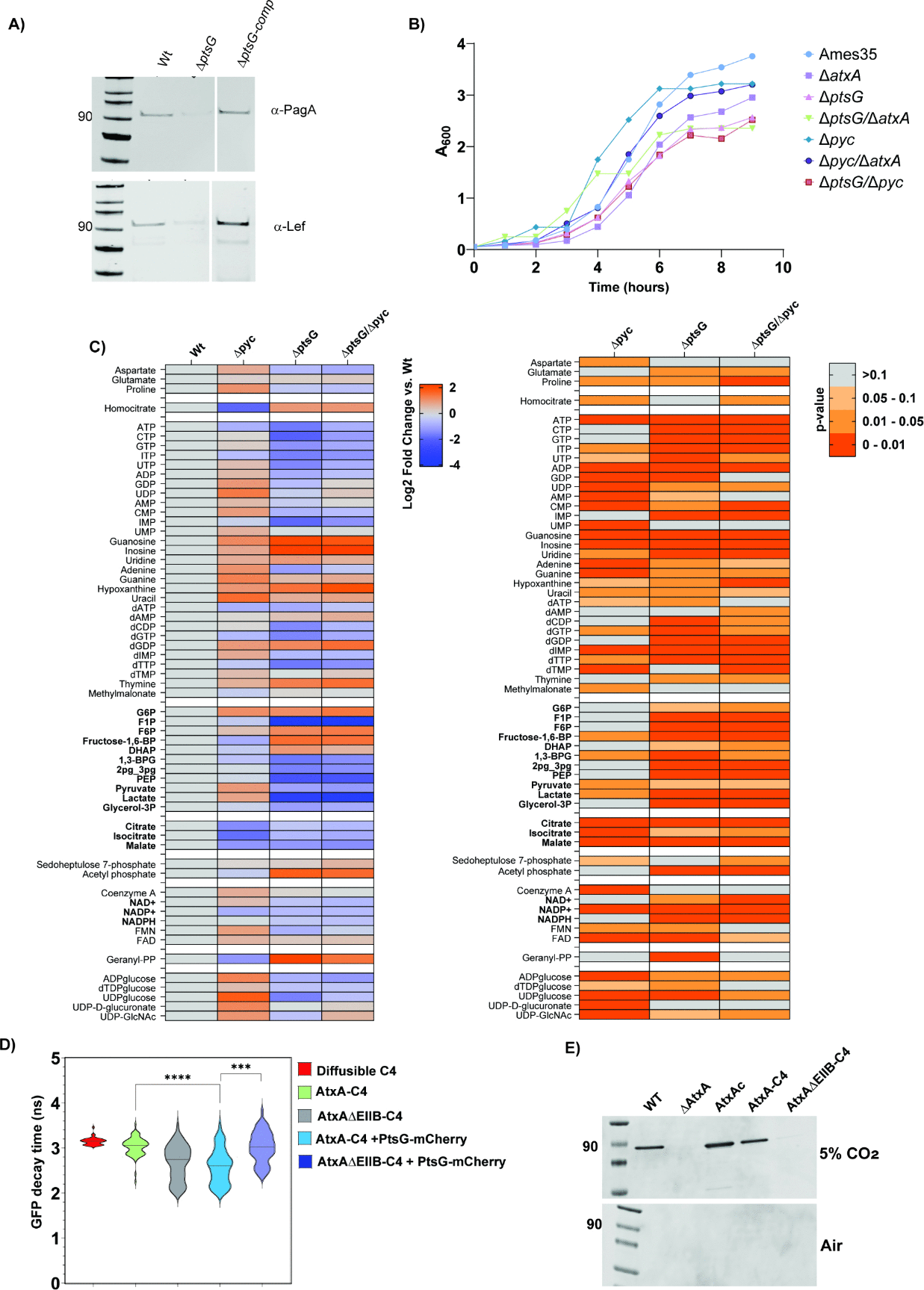
**

**A)** Immunoblotting for anthrax toxin components (PagA and Lef) in *B. anthracis* WT, Δ*ptsG*, and its complemented strain (Δ*ptsG*-Comp). Supernatant volumes were normalized against total cellular protein for comparative analysis. Representative graph of n=2 experiments.

**B)** Growth kinetics of all *B. anthracis* strains under toxin-producing conditions (glucose + 5% CO_2_). Each data point represents the average of three replicates. Representative image of n=2 experiments.

**C)** Heatmap showing the relative abundance of significantly altered metabolites in Δ*ptsG*, Δ*pyc*, and Δ*ptsG*/Δ*pyc* mutants compared to their respective abundance in *B. anthracis* WT strain grown under toxin-producing conditions (glucose + 5% CO_2_). The left heatmap shows the log2 fold change in abundance as a color gradient from blue (low abundance) to red (high abundance). The right heatmap corresponds to the p-value of each data point on the left heatmap, represented as a color gradient from grey (p > 0.1, insignificant) to orange (p = 0-0.01, highly significant).

**D)** Representative FRET-FLIM based interactions in strains expressing different FRET partners. The violin/distribution plot represents the median GFP decay profile (in nanoseconds, ns) GFP variant C4 in the respective strains. The graph represents n=2 experiments, * indicates p-value.

**E)** Immunoblotting for PagA showing effects of loss of the EIIB domain of AtxA (AtxA-ΔEIIB-C4) on anthrax toxin expression. Having AtxA variants expressed in the background of *B. anthracis ΔatxA* strains, with plasmid pAMY expressing the respective AtxA variants. Representative image of n=3 experiments.
