# Supplementary material for: Environmental regulation of toxin production in Bacillus anthracis
Source: PLoS Pathog. 2025 Dec 1;21(12):e1013587. doi: 10.1371/journal.ppat.1013587 (PMC12680359; doi:10.1371/journal.ppat.1013587)
Supplement: S5 Fig — The two units of the AtxA dimer are shown in rainbow and golden colors. B) Zoomed image of the interaction between C402 of one unit (rainbow-colored) and H379 of the other unit (golden-colored). The electrostatic interaction zones of both residues are shown as white clouds around them. C) Virulence of parent and PTSglu and Pyc mutants. A. Survival curves of mice infected sub-cuteneously (s.c.) with spores B. anthracis are shown. C57BL/6J mice were injected s.c. with 8X106 CFU of the Wt (red line; n = 5), ΔptsG (green; n = 5), and ΔptsG/ Δpyc (black; n = 5). (DOCX) [file ppat.1013587.s005.docx]

**S5 Fig.**

**
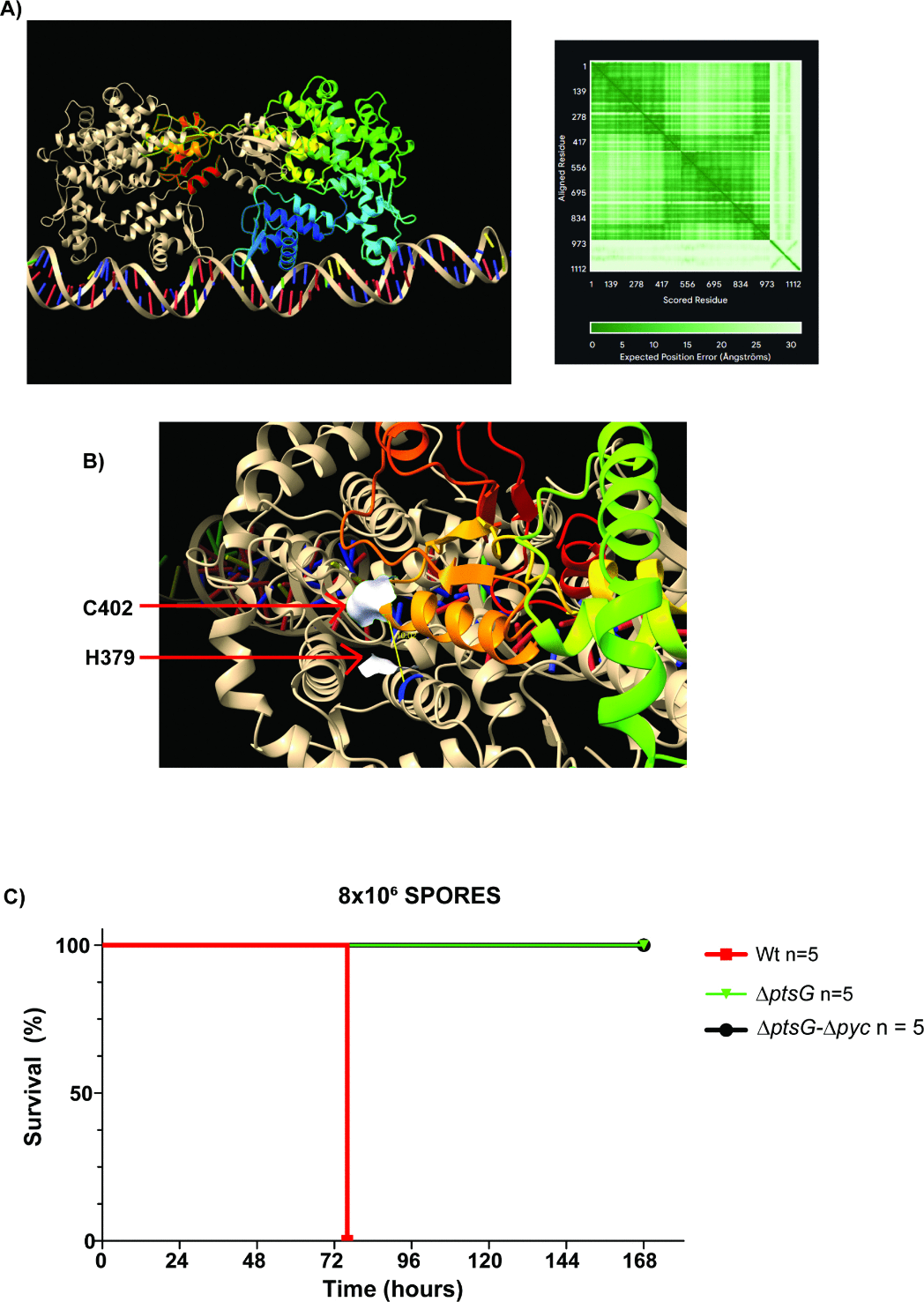
**

**A, B)** Alpha-fold predicted structure of WT AtxA dimer with the *pagA* promoter carrying AtxA-binding elements. **A)** The two units of the AtxA dimer are shown in rainbow and golden colors; **B)** Zoomed image of the interaction between C402 of one unit (rainbow-colored) and H379 of the other unit (golden-colored). The electrostatic interaction zones of both residues are shown as white clouds around them.

**C)** Virulence of parent and PTS^glu^ and Pyc mutants. A. Survival curves of mice infected sub-cuteneously (s.c.) with spores *B. anthracis* are shown. C57BL/6J mice were injected s.c. with 8X10^6^ CFU of the Wt (red line; n = 5), Δ*ptsG* (green ; n=5) , and Δ*ptsG/* Δ*pyc* (black ; n=5).
