# Supplementary material for: Environmental regulation of toxin production in Bacillus anthracis
Source: PLoS Pathog. 2025 Dec 1;21(12):e1013587. doi: 10.1371/journal.ppat.1013587 (PMC12680359; doi:10.1371/journal.ppat.1013587)
Supplement: Attachment — Submitted filename: Response to Reviewers.pdf [file ppat.1013587.s009.pdf]

Title: *Environmental Regulation of Toxin Production in Bacillus anthracis*

Manuscript ID: PPATHOGENS-D-25-02437

Response to Reviewers

We thank the Editor(s) and all three reviewers for their thorough and constructive evaluation of our work. We are encouraged that all reviewers found the study significant, technically strong, and of broad relevance. We have carefully considered each comment and revised the text, figures, and supplementary materials accordingly. Where new experiments were suggested but were not feasible within the scope of the current study, we have provided clarifications, supporting literature, and a scientific rationale instead.

Below, we respond to each comment point-by-point. Reviewer comments are **bold**, and our responses follow them in chronology.

Reviewer #1

1. **Difficulty identifying proteins in the supplemental table and gene names missing.**
   We thank the reviewer for pointing this out. The Supplementary table-2 has been updated to include both gene names with their description for all AtxA-interacting proteins for clarity. This should make it easier to interpret the dataset. The total number of identified proteins remains unchanged, and this addition and clarification does not affect the results or conclusions.

2. **The number of interacting proteins seems high; please provide support for this being typical for transcription factors.**
   We thank the reviewer for this valuable comment. We have clarified in the Discussion that the large number of co-purifying proteins is consistent with previous affinity-based studies of bacterial transcription regulators, which often recover transcription/translation and metabolic partners (Ref 13, 15, and 37). The revision has been made between line 399-402.

3. **Was a reciprocal pulldown with His-tagged PtsG attempted?**
   We thank the reviewer for this question. A reciprocal pulldown with His6-tagged PtsG was indeed attempted; however, because PtsG is a membrane-embedded permease, recovery was unsuccessful even after ultracentrifugation and mild detergent solubilization. We, therefore, relied on complementary approaches, such as co-affinity purification, FLIM-FRET interaction analysis, and phenotypic assays, which together robustly support the AtxA-PtsG interaction.

4. **Are there other PTS components beyond PtsG that might interact with AtxA?**
   We thank the reviewer for this thoughtful point. Additional PTS-related proteins (such as PtsI and HPr) were indeed detected in the interactome; however, their spectral abundance was variable across replicates and conditions (air vs. CO_2) and fell below our defined confidence threshold. Moreover, some of these proteins were also detected in negative-control pulldowns, suggesting possible nonspecific association. For these reasons, only high-confidence interactors such as PtsG and Pyc are discussed in the main text, while the complete dataset is presented in Supplementary Figure- 3 and Supplementary Table- 2.

5. **Please expand discussion to integrate with earlier models (e.g., Bier et al., 2020) and comment on whether transcriptional regulation of atxA itself plays a role.**
   We thank the reviewer for this helpful suggestion. We have expanded the Discussion to integrate our findings with earlier models, particularly those proposed by Bier et al. (2020). Our results showing a direct interaction between AtxA and PtsG complement these earlier observations that PTS components may influence atxA expression indirectly through unknown regulators. Together, these findings suggest that both transcriptional and post-translational mechanisms contribute to AtxA regulation in Bacillus anthracis, providing a more integrated view of how carbon-source sensing connects to toxin control. The revised text has been added in the Discussion (new lines 441-446).

Reviewer #2

1. **Line 194 – incorrect figure reference.**
   Corrected to Figure 1E.

2. **Clarify that glucose was present in both conditions when comparing AtxA interactome.**
   Sentence on line 197 (new line 211-212) revised to: "Therefore, we compared the AtxA interactome in bacteria grown with glucose in the presence or absence of 5 % CO_2.".

3. **Were proteins involved in AtxA phosphorylation identified in the interactome?**
   We thank the reviewer for this insightful question. We examined the complete AtxA interactome for potential kinases or phosphatases that could participate in AtxA phosphorylation. Under 5 % CO_2 conditions, two metabolic kinases, 1) Aspartokinase (DapG1) and 2) Ribose-phosphate pyrophosphokinase (Prs), were detected among the low-abundance interactors (Supplementary Table- 2, (and related data summarized in Supplementary Figure- 3)). However, these enzymes are primarily involved in amino acid and nucleotide biosynthetic pathways, respectively, and are not known to mediate protein phosphorylation. No canonical histidine kinases predicted to modify AtxA were identified above our confidence threshold. This observation supports our model that AtxA activity is

modulated indirectly through PTS-linked phosphorelay mechanisms rather than via direct kinase interaction.

4. **Are FRET-FLIM image scales identical across panels (Figure 3C)?**
   We thank reviewer for pointing this out. The scale bars are identical across all panels and have been added in the figure and legend.

5. **Could FRET-FLIM have been performed in presence of CO_2/bicarbonate?**
   We thank the reviewer for this comment. In fact, the FLIM experiments were conducted under bicarbonate / 5 % CO_2 conditions to mirror the toxin-inducing environment. We have clarified this in the Methods (new line 651) and in the Figure- 3C legend (new line 882-883)

6. **Could glucose uptake be measured in the ΔptsG mutant?**
   We appreciate this thoughtful suggestion. While direct glucose-uptake assays with the *ΔptsG* strain were not performed, our metabolomic profiling (Figure 2A; Supplementary Figure- 4C) demonstrates depletion of lower glycolytic intermediates such as phosphoenolpyruvate and pyruvate, consistent with reduced glucose uptake and PTS activity. Together with the markedly reduced toxin gene expression (Figure- 4A) and otherwise near-normal growth under glucose + 5 % CO_2 (Supplementary Figure- 4B), these findings strongly support that PtsG serves as the principal glucose permease linking carbon metabolism to toxin induction.
   We have added a statement in the Discussion to address this question on new line 390-392.

7. **Minor typographical corrections**
   All have been corrected.
   - Line 264: replace CRH motif to CIXR motif.
   - Line 484: average AD600 to average OD600.
   - Line 555: update to uptake.
   - Line 520 (new line 555 and 575): Full reference replacing DOI placeholder has been added.
   - Line 824: “in” removed from Figure legend 2A.

8. **Did the authors test germination efficiency of the ΔptsG and ΔptsG/Δpyc spores?**
   We thank the reviewer for this important question. Although we did not include germination data, we believe the attenuation of the *ΔptsG* and *ΔptsG*/*Δpyc* strains cannot be attributed to a germination defect. Both mutants displayed near-normal vegetative growth under glucose + 5% CO_2 conditions (Supplementary Figure- 4B), and the attenuation persisted even when vegetative bacteria were used for infection (Figure- 5A). These observations indicate that the reduced virulence arises from a failure to establish

infection post-germination, likely due to impaired toxin induction under host-relevant conditions. We have clarified this point in the Discussion on new line 432-435.

9. **Could mouse diet influence infection outcomes?**
   We thank the reviewer for this insightful comment. All animal experiments were conducted under the approved NIH IACUC protocol LPD9E, which includes standard husbandry and feeding conditions. Mice were maintained on normal chow with no specialized diet. We agree that host metabolic status could influence bacterial virulence, and this is an interesting aspect that merits future investigation. However, in our current study all groups were maintained under identical conditions, so dietary differences are unlikely to have contributed to the observed outcomes.

10. **Figure 6 does not show Pyc-AtxA interaction; please explain.**
   We thank reviewer for this detailed observation. The interaction between AtxA and Pyc was detected under both air and CO_2 conditions (Supplementary Table- 2), suggesting that it is not specific to the toxin-inducing CO_2 environment. For this reason, Pyc was not included in the schematic model (Figure 6), which focuses on CO_2-dependent, PtsG-specific interactions driving toxin induction. We interpret the AtxA-Pyc interaction as likely reflecting broader metabolic coupling rather than direct CO_2-specific regulation.

Reviewer #3

1. **Could the AtxA EIIB C402 site be phosphorylated, analogous to Mga in *S. pyogenes*?**
   We appreciate this insightful comparison. Based on AlphaFold modeling and previous structural studies (Hammerstrom et al., 2015), the cysteine at position 402 Seem critically important for AtxA activity. In our AlphaFold analysis C402 lies adjacent to H379, suggesting potential for redox or phospho-modulation. While we did not directly test for phosphorylation at this site, our mutational data (C402A/G) indicate that the residue is critical for AtxA function. Following reviewer's suggestion, we discuss the analogy to Mga (Woo et al., 2024) and MtlR (Joyet et. al. 2010) in the revised Discussion on new line 406-412.

2. **Regarding the attenuation of the ptsG deletion strains – while I appreciate that the subcutaneous injection of vegetative cells into a mouse allows one to bypass the germination step required for spores, I don't completely follow that one can conclude that PTS-Glu is required as a sensor for toxin production vs required for bacterial survival and replication in the host as a result of its role in carbon metabolism. Are the authors concluding that toxin production is key based on the timing of mice**

succumbing to infection by vegetative cells? Would vegetative cells that constitutively produce toxin bypass the need for PtsG??

We thank the reviewer for this detailed and thoughtful question. Our data indicate that the attenuation of the *ΔptsG* and *ΔptsG/Δpyc* mutants is primarily due to the loss of AtxA-dependent toxin induction rather than a defect in basic metabolism or germination. Both mutants grew comparably to the wild type under glucose + 5 % CO_2 (Supplementary Figure 4B), and attenuation persisted even when vegetative bacteria were used for infection (Figure 5A), showing that the defect arises post-germination. Deletion of *ptsG* caused a ~90 % reduction in toxin gene expression (Fig. 4A), that was fully restored in the complemented strain. Moreover, overexpression of the phosphomimetic AtxA(H199D) mutant in a *ΔptsG/ ΔatxA* background did not rescue toxin expression (Figure- 4B), demonstrating that PtsG is required upstream of AtxA (possibly as a sensor to activate AtxA) and that phosphorylation mimicry alone is insufficient for activation. Together with the depletion of lower glycolytic intermediates and phosphoenolpyruvate observed in the metabolomic data (Figure- 2A; Supplementary Figure- 4C), these results support a model in which PtsG functions primarily as a sensor linking glucose flux to AtxA activation and toxin production, rather than as a transporter required for bacterial growth.

Also, constitutive toxin expressions would be expected to be extremely lethal and may bypass the need for PtsG-mediated activation, but this specific experiment was beyond the scope of the current study and is being explored separately. This interpretation is now reflected in the revised Discussion and related clarifications made in response to other reviewer comments.

3. **Minor textual issues (title of section, “True/False” panel, incomplete sentence).**

   All have been corrected.

   - Figure- 1C and Supplementary Figure-1A, 1C now includes details as up-regulated and down-regulated gene sets, instead of “True/False”, to denote significantly enriched KEGG pathways (padj < 0.05).
   - The incomplete sentence in line 420 has been corrected for clarity.
   - Line 99: change section title to “Anthrax toxin production is dependent on a central carbon metabolism gene.”
   - Figure- 1C legend (page 20): Then figure has been modified to address this concern.
   - Line 421 (incomplete sentence): The sentence has been modified.

We are grateful for the reviewers’ thoughtful feedback, which has improved the clarity and contextual strength of the manuscript. We hope these revisions satisfactorily address all concerns.
